# The prognostic value of the stem-like group in colorectal cancer using a panel of immunohistochemistry markers

**DOI:** 10.18632/oncotarget.3497

**Published:** 2015-03-26

**Authors:** Chee Wee Ong, Pei Yi Chong, Darragh G. McArt, Jason Yongsheng Chan, Hwee Tong Tan, Alan Prem Kumar, Maxey C. M. Chung, Marie-Véronique Clément, Richie Soong, Sandra Van Schaeybroeck, David J. J. Waugh, Patrick G. Johnston, Philip D. Dunne, Manuel Salto-Tellez

**Affiliations:** ^1^ Centre for Cancer Research and Cell Biology, Queen's University Belfast, Northern Ireland; ^2^ Cancer Science Institute of Singapore, National University of Singapore, Singapore; ^3^ Department of Pharmacology, Yong Loo Lin School of Medicine, National University of Singapore, Singapore; ^4^ Curtin Health Innovation Research Institute, Biosciences Research Precinct, School of Biomedical Sciences, Faculty of Health Sciences, Curtin University, Western Australia, Australia; ^5^ Department of Biological Sciences, University of North Texas, Denton, Texas, United States of America; ^6^ Department of Biochemistry, Yong Loo Lin School of Medicine, National University of Singapore, Singapore; ^7^ National University of Singapore Graduate School for Integrative Sciences and Engineering, National University of Singapore, Singapore; ^8^ Department of Medical Oncology, National Cancer Centre Singapore, Singapore

**Keywords:** colorectal, carcinoma, biomarkers, cancer stem-cell, immunohistochemistry

## Abstract

Colorectal cancer (CRC) is the second leading cause of cancer-related deaths in the Western world. It is becoming increasingly clear that CRC is a diverse disease, as exemplified by the identification of subgroups of CRC tumours that are driven by distinct biology. Recently, a number of studies have begun to define panels of diagnostically relevant markers to align patients into individual subgroups in an attempt to give information on prognosis and treatment response. We examined the immunohistochemical expression profile of 18 markers, each representing a putative role in cancer development, in 493 primary colorectal carcinomas using tissue microarrays. Through unsupervised clustering in stage II cancers, we identified two cluster groups that are broadly defined by inflammatory or immune-related factors (CD3, CD8, COX-2 and FOXP3) and stem-like factors (CD44, LGR5, SOX2, OCT4). The expression of the stem-like group markers was associated with a significantly worse prognosis compared to cases with lower expression. In addition, patients classified in the stem-like subgroup displayed a trend towards a benefit from adjuvant treatment. The biologically relevant and poor prognostic stem-like group could also be identified in early stage I cancers, suggesting a potential opportunity for the identification of aggressive tumors at a very early stage of the disease.

## INTRODUCTION

To date, the strongest prognostic value is derived from the tumor node metastasis system of staging (TNM). Patients with stage I disease have the best prognosis, with over 90% surviving past five years. These survival rates decrease to less than 5% for patients with advanced stage IV disease. The heterogeneity of colorectal cancers (CRC) at both the molecular level and in terms of prognosis is apparent; even within each stage, as there exist groups of patients with variation in both overall survival and response to either standard of targeted treatment strategies. The addition of clinicopathological markers such as tumor grade, invasion of lymph nodes, blood vessels or perineural invasion can further be used to identify poor prognostic subgroups of patients within each stage, but no definitive molecular signature has yet been attributed to these subgroups.

The development of meaningful subgrouping based on gene expression data, which related to prognosis, was first pioneered in breast cancer by two landmark papers in 2000 and 2001 [1, 2]. These paradigm-shifting publications emphasized how tumor specific gene expression data, through the use of high throughput microarray technology, could be used to classify patients based on the biology underlying their disease. In the last decade a number of studies have been published outlining prognostic gene signatures in colorectal cancer using various high throughput microarray platforms [3–5]. However, as these prognostic signatures were purely generated for their prognostic value they failed to address the driving biology behind each individual cancer or to relate that to potential treatment strategies. Given the extreme variation in prognosis, particularly in early stage colorectal cancer, these signatures were initially aimed at informing clinical decision around whether adjuvant treatment following surgery would provide any benefit over watchful surveillance. Nevertheless, the publication of three recent studies sought to set a new paradigm for the classification of colorectal cancer based on gene expression profiling [6–8]. While each of these studies generated independent transcriptional signatures, a common theme was the emergence of a stem cell subtype; which defined the poor prognostic group in stage II CRC. The importance of the prognostic value of this stem cell subtype was subsequently validated in the CRC consensus analysis presented at the American Society of Clinical Oncology (ASCO) meeting in 2014 [9].

Another notable characteristic of these studies were that apart from defining the biology driving disease progression, they also attempted to develop clinically useful IHC panels to assign patients to each subtype. To date, these IHC panels have yet to be fully defined or validated in retrospective cohorts or prospective trial setting. Hence, in this study, we analyze a well-characterized retrospective CRC cohort in order to test the prognostic value of the stem-like and inflammatory subtypes, using unsupervised clustering in relation to overall survival. This was initially examined in stage II cancers, followed by the other cancer stages. Moreover, our findings are further tested by patient stratification into either in treated and untreated groups, with the aim of validating the prognostic value of the identified subgroups in CRC using routine standard IHC methods.

## RESULTS

### Unsupervised hierarchical clustering of stage II CRC

**Figure 1:**
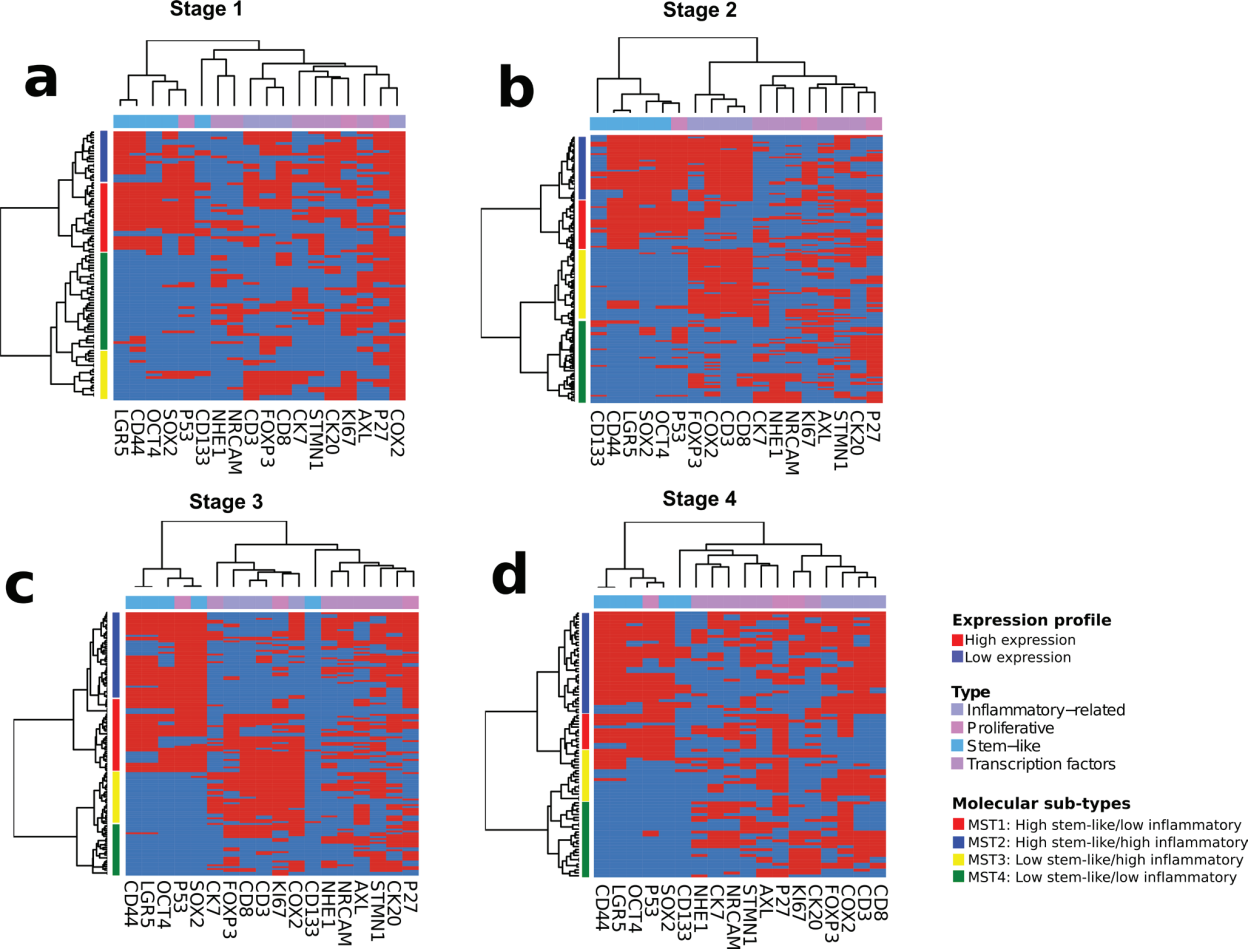
Unsupervised hierarchical clustering analysis of immunohistochemical staining profile Heatmaps showing appearance of distinct patients clusters in Stage I **a.** II **b.** III **c.** and IV **d.** colorectal cancer cases.

Consensus unsupervised analysis of the immunohistochemistry scoring revealed proteins with co-expression patterns that grouped into two distinct clusters and one heterogeneous cluster (Figure 1b). Expression cluster 1 contained predominately markers associated with stem-like characteristics; CD44, LGR5, SOX2 and OCT4, while cluster 2 contained inflammatory and immune associated markers; CD8, CD3, COX2 and FOXP3. Cluster 3 was a more heterogeneous group containing markers associated with a wider range of characteristics. Given the clear biology emerging at this stage, we focused on the identified distinct clusters 1 and 2. Expression of either a high stem-like or inflammatory pattern was not mutually exclusive and resulted in the appearance of four clearly distinct patient clusters. Using these clusters, we categorized the patients into one of four molecular sub-types (MST1–4) based on their expression pattern of either the stem-like markers or the inflammatory markers. These subtypes were MST1; high stem-like/low inflammatory, MST2; high stem-like/high inflammatory, MST3; low stem-like/high inflammatory, MST4; low stem-like/low inflammatory (Figure 1).

### Prognostic relevance of identified molecular sub-types in stage II CRC

**Figure 2:**
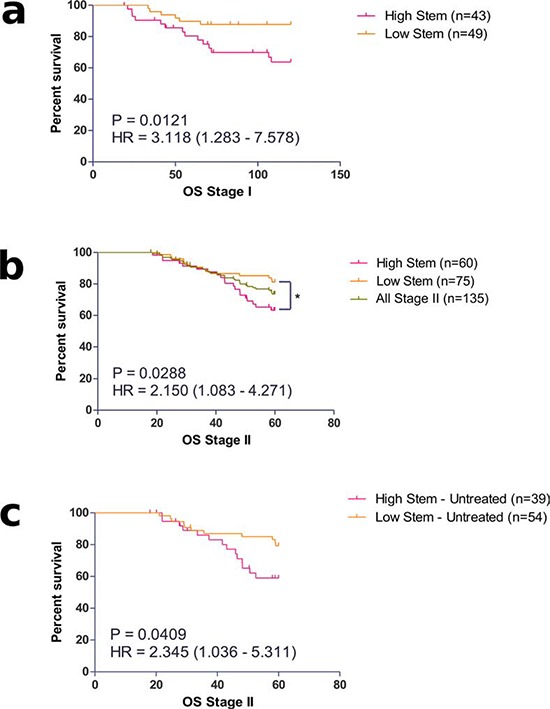
**Prognostic value of identified stem-like molecular sub-groups in a. Stage I and b. II colorectal cancer patients.** The prognostic value of the stem-like classifier was also observed in **c.** Stage II patients who received no adjuvant treatment.

To investigate the clinical relevance of our identified subtypes, we carried out survival analysis using overall survival limited to five years in our stage II cohort. While there was no significant difference in survival between MST1 to 4 (Supplementary Figure S1), there appeared to be a trend towards a worse prognosis in MST1 and MST2, which both shared high expression of stem-like markers, compared to MST3 and MST4. We then re-classified the patients into high or low groups for either stem-like or inflammatory markers for survival analysis. A significantly worse prognosis was found in patients categorized in the high stem-like group compared to those with lower expression (Supplementary Table S1, *p* = 0.0288). The separation of patients using this stem-like classifier (Figure 2b) resulted in a group with a hazard ratio (HR) of 2.150 (CI 1.083 – 4.271). In contrast, overall survival was unaffected by classification using the inflammatory panel of markers.

### Effect of adjuvant treatment on prognosis of identified subgroups in stage II CRC

Within our stage II cohort, we have patients who were either treated with adjuvant chemotherapy (30%) or untreated (70%). Although the treatment was heterogeneous, it was predominantly fluorouracil based (5FU). Analysis of the patients who received no adjuvant treatment confirmed the true prognostic value of the stem-like classifier (Figure 2c), with an increased HR of 2.345 (CI 1.036 – 5.311; *p* = 0.0409) in the high-stem patients compared to the low-stem sub-group. More importantly, analysis of the stem-like classifier in the treated-only group failed to show any prognostic value to the classifier (Supplementary Figure S2), suggesting that treatment of patients in the poor prognostic high stem-like group might improve their overall survival.

To further test this hypothesis, patients displaying a high stem-like classification appeared to show a trend towards a benefit from treatment with adjuvant chemotherapy as highlighted by an improved overall prognosis in the treated group compared to the untreated group, although this finding was not significant (Supplementary Figure S3).

### Identification of stem-like and inflammatory associated subgroups in later stage CRC

Following identification of molecular subtypes in early stage patients, we carried out a similar unsupervised clustering of stage III and stage IV patients in our cohort using the same classifiers for both stem-like and inflammatory markers (Figure 1c and 1d). This approach revealed the presence of all four of our stage II molecular subtypes, in each of the later stages, alongside the individual stem-like or inflammatory groups. Although the presence of both the stem-like and the inflammatory groups could be identified in stage III and IV patients in our cohort, there was no prognostic value to our classifier in these advanced stage cases.

### Prognostic value of the stem-like classifier in pre-invasive stage I colorectal patients

Next, we sought to examine whether the stem-like group, which has prognostic effect in stage II, may also identify patients with poor outcome in the pre-invasive early disease population. We performed survival analysis comparing the stem-high against the stem-low group in the stage I cases. As these patients have a relatively good prognosis compared to stage II patients we used a 10-year follow up for this analysis (Figure 2a). We observed a significantly worse overall prognosis for patients classified in the stem-high group with an increased risk of death over 3 times that of the low-stem group (HR = 3.118, CI 1.283 – 7.578, *p* = 0.0121).

## DISCUSSION

Even though numerous studies have consistently showed evidence of different subtypes outlining the heterogeneity nature of colorectal cancer [6, 10, 11], the integration of these gene expression profiles in the clinical context still require extensive validation. Using our well-characterized retrospective cohort of patients with extensive survival follow-up, we aimed to define markers of clinically relevant and diagnostically useful subgroups in colorectal cancer. As with a number of other studies, we initially focused on the stage II patients within our cohort. Using unsupervised consensus-based clustering, we found that our dataset subdivided into three general clusters based on their IHC profiles (Figure 1, Supplementary Tables S2). Although one of these clusters represented biology which was heterogeneous in nature, it was interesting that the other two clusters were underpinned by clearly distinct driving biology, namely, a subgroup showing high expression of inflammatory-related factors (CD3, CD8, COX-2 and FOXP3) and a subgroup showing high expression of stem-like factors (CD44, LGR5, SOX2 and OCT4). The identification of these two biologically diverse inflammatory-related and stem-like clusters have been reported in a number of other studies through transcriptional profiling of colorectal cancer [6].

The high expression of the inflammatory-related factors CD3, CD8 and FOXP3 has been shown in a number of other studies [12–14]. FOXP3 has been reviewed to be associated with either good or neutral prognostic effect in colorectal cancer [12]. Thus, it has been suggested that the favorable prognostic effect of FOXP3 may be due to the ability to suppress tumor-promoting inflammatory responses to gut microbes [13]. It has been hypothesized that increased inflammation could either provide a favorable environment for the dissemination of tumor cells or an indication of protective host responses [14]. The improved survival associated with CD3 and CD8 expression could be an effect of micrometastatic suppression induced by a systemic immunosurveillance mechanism [15]. The role of COX2 in the immune tumor microenvironment in colon cancer is well-defined and the uses of inhibitors, such as aspirin, in prevention of hereditary cancers of the colon are commonly accepted [16, 17].

In normal tissue homeostasis, the regulation of cellular renewal is carefully maintained by stem-cells and progenitor cells. The loss of this mechanism may be the underlying cause of deregulated cell proliferation leading to cancer development [18]. Both CD44 and LGR5 are widely recognized as key regulators of the stem cell phenotype, particularly in colorectal cancer [19, 20]. While the transcription factors SOX2 and OCT4 are increasingly reported to contribute to cancer progression through their functional control of cell self-renewal and the pluripotent state [21, 22]. Although both CD133 and p53 appeared to cluster within the stem-like subgroup, we chose to leave these factors out of further analysis. This was due to the overall low levels of staining observed for CD133 within our TMA (Table 2) and the current uncertainty about using p53 staining as a surrogate for mutation identification. Although given the role for both CD133 and p53 in cell self-renewal these finding do justify further investigations into the relationship of these markers within a stem-like signature.

Our finding, which shows that stem-like phenotype is associated with worse overall survival in early stage colorectal cancer, is very similar to the findings of a number of independently published transcriptional based classification studies [8–10]. While these other studies have proposed IHC markers to define their identified subtypes, using our retrospective cohort, we have shown that our panel of CD44, LGR5, SOX2 and OCT4 can similarly identify this poor prognostic subgroup. We see a clear prognostic value of our stem-like classifier in the overall stage II population (Figure 2b) and more so in the untreated patients, where the true prognostic value can be identified (Figure 2c). It is interesting however, that when patients in the adjuvant treated group are analyzed, the stem-like classifier is no longer prognostic (Supplementary Figure S2). Further analysis revealed that there was a clear trend for improved survival in the high stem-like subgroup following the addition of adjuvant treatment, although this effect was not significant, while there was no trend for improved survival in the low-stem group (Supplementary Figure S3). This trend towards a benefit from treatment in the otherwise poor prognostic group displays similarities to the study by Sadanandam et al., where they found no association between their stem-like subtype and DFS in patients receiving adjuvant therapy. Similar to the data presented here, when the untreated patients were selected individually the stem-like group was found to have a significant association with lower survival (Figure 2c). Given that a large proportion of stage II patients who are given adjuvant therapy do not gain any benefit from this treatment, this finding warrants further investigation, as current decisions on whether to offer adjuvant chemotherapy still rely on a number of other classical prognostic factors.

Although stage I patients currently have a very favorable outcome, with a 5-year survival rate of over 90%, we found that the stem-like IHC panel could identify a subgroup with a poor overall survival rate (Figure 2a). Currently the proportion of patients diagnosed at stage I is low, approximately 9% in the UK, but given the recent expansion of colorectal cancer screening programs in the UK and worldwide, patients identified at this early stage will no doubt increase from its current rate [23]. Having a routine method of testing the projected increased numbers of early stage cases for traits associated with disease progression represents an opportunity for early intervention. Evidence for the existence of transcription profiles similar to those observed in poor prognostic groups of established colorectal cancer have been found in early serrated adenoma lesions, with suggestions that the driving biology found in the stem-like tumors may already be established in precursor lesions [7]. This latter point highlights a need for the expansion of similar studies to archived polyp collections for further investigation.

In summary, our results clearly reflect the molecular heterogeneity of colorectal cancer and are in agreement with observations in gene expression profiling studies [6, 10, 11]. Our findings also address the relevance of inflammatory and stem-like molecular subgroups in colorectal cancer. More importantly, the difference in the survival outcome between the different subtypes suggest that colorectal cancer management could be potentially improved by the inclusion of biomarker information [24, 25]. In addition the poor-prognostic stem-like subgroup identified in this cohort appears to show a trend for improved survival rates following adjuvant chemotherapy, which may aid in selecting patients who will derive benefit from treatment. The results from our study may help shed some light on identifying this particular subtype for targeted clinical intervention, particularly at the very early stage, where there is only a small subgroup of patients who will derive any benefit from the addition of adjuvant chemotherapy following surgery.

## MATERIALS and METHODS

### Patient cohort and selection of immunohistochemical markers

The patient cohort in this study comprises of 493 CRC cancer patients that underwent primary surgery for the disease from 1990 to 1999 in the National University Hospital of Singapore. For this study, formalin-fixed and paraffin-embedded tissue blocks were retrospectively collected from the Department of Pathology. Clinical and pathological data were extracted from the medical records for the purpose of the study. The procedure for this research was approved by the ethics committee of the National University of Singapore. Patient characteristics are summarized in Table 1. The information retrieved included gender, age, tumor size, tumor stage (AJCC), histological grade, vascular invasion, perineural invasion, and lymphatic invasion. Patients were staged according to the AJCC TNM classification and were monitored for relapse of disease and death. Survival time was measured from the date of diagnosis until the date of death. Patients alive after 60 months was considered censored.

**Table 1:** Summary of patient characteristics (*n* = 493)

Clinical parameters	Number and percentage of cohort (total *n* = 493)
**Age**	
*< 65*	247 (50%)
*≥ 65*	246 (50%)
**Gender**	
*Male*	240 (48%)
*Female*	257 (52%)
**AJCC stage**	
*1 and 2*	262 (53%)
*3 and 4*	231 (47%)
**Ethnicity**	
*Chinese*	429 (87%)
*Non-Chinese*	68 (13%)
**Tumor site**	
*Colon*	109 (22%)
*Rectal*	388 (78%)
**Tumor size**	
*< 5 cm*	194 (56%)
*≥ 5 cm*	303 (44%)
**Tumor differentiation**	
*Poor*	59 (12%)
*Well and moderate*	438 (88%)
**Metastasis status**	
*Positive*	121 (25%)
*Negative*	378 (75%)
**Vascular invasion**	
*Present*	50 (10%)
*Absent*	447 (90%)
**Perineural invasion**	
*Present*	41 (8%)
*Absent*	456 (92%)
**Lymphatic invasion**	
*Present*	19 (4%)
*Absent*	478 (96%)

For this study, a panel of 18 immunohistochemical markers were analyzed (Table 2). These markers were broadly selected and categorized by their putative role in development of colorectal cancer such as such having tumor suppressive, oncogenic, proliferative, inflammatory and stem-like properties.

**Table 2:** Summary of characteristics and expression frequency of markers, conditions used for immunohistochemistry and previous publications using the same patient cohort

Marker	Clone	Manufacturer	Dilution	Expression frequency (*N* = 493)^*^	Localisation	Previous publication
AXL	HPA037422	Sigma Aldrich, MO, USA	1:1000	219 (65%)	Cytoplasmic	P Dunne et al., Clin Can Res (2014) [27]
CD3	LN10	Novocastra, Newcastle, UK	1:1000	246 (50%)	Nuclear	Unpublished
CD8	11F1	Novocastra, Newcastle, UK	1:40	256 (52%)	Nuclear	Unpublished
CD133	AC133	Miltenyi Biotech, CA, USA	1:10	60 (12%)	Membrane	CW Ong et al., Modern Path (2010) [26]
CD44	SFF-304	Bender MedSystems, CA, USA	1:250	230 (47%)	Cytoplasmic	Unpublished
CK7	OV-TL-12/30	Santa Cruz, CA, USA	1:500	159 (32%)	Cytoplasmic	CW Ong et al., Modern Path (2010) [26]
CK20	KS20–8	Dako, Denmark	1:500	226 (42%)	Cytoplasmic	CW Ong et al., Modern Path (2010) [26]
COX2	M-19	Santa Cruz, CA, USA	1:250	351 (71%)	Cytoplasmic	CW Ong et al., Modern Path (2010) [26]
FOXP3	236A/E7	Abcam, MA, USA	1:250	234 (47%)	Nuclear	Unpublished
Ki67	SP6	Abcam, MA, USA	1:200	183 (37%)	Nuclear	CW Ong et al., Modern Path (2010) [26]
LGR5	EPR3065Y	Novus Biologicals, CO, USA	1:300	245 (70%)	Cytoplasmic	Unpublished
NHE1	PRS4377	Sigma Aldrich, MO, USA	1:100	335 (68%)	Membrane	Unpublished
NRCAM	ab24344	Abcam, MA, USA	1:500	272 (55%)	Cytoplasmic	JY Chan et al., Cancer Sci. (2011) [28]
OCT4	H-143	Santa Cruz, CA, USA	1:1000	279 (56%)	Cytoplasmic	CW Ong et al., Modern Path (2010) [26]
p27	SX53G8	Abcam, MA, USA	1:500	299 (61%)	Nuclear	CW Ong et al., Modern Path (2010) [26]
p53	D-07	Dako, Denmark	1:250	245 (49%)	Nuclear	CW Ong et al., Modern Path (2010) [26]
SOX2	57CT23.3.4	Abcam, MA, USA	1:1000	235 (47%)	Cytoplasmic	CW Ong et al., Modern Path (2010) [26]
STMN1	3352	Cell Signaling, MA, USA	1:250	235 (48%)	Cytoplasmic	HT Tan et al., J Proteome Res. (2012) [29]

* Number of actual cases used in previous studies may differ from the total number of cases examined due to availability of clinical information, loss of cores or lack of tumor materials available for pathological scoring purposes during the time of study.

### Tissue microarray construction and immunohistochemistry

The details of construction of the tissue microarray has been previously described [26]. Briefly, representative areas of tumor were initially marked on hematoxylin and eosin stained slides by a pathologist. These areas were taken from the center of the colorectal tumor and were subsequently cored from the donor tissue block using a 0.6 mm diameter cylinder. The cylinder cores were then transplanted into a recipient paraffin block. These processes were carried out using an ATA-100 tissue arrayer (Chemicon International, Temecula, CA, USA). Three cores were taken from tissue blocks from each of the patients. A total of 10 tissue microarray blocks, arrayed in triplicates, were constructed for this study. Consecutive 5-μm sections were then placed on polylysine-coated slides for immunohistochemical analyses.

Heat-mediated antigen retrieval methods were used for all the immunohistochemical markers; these were carried out using either the MicroMED TT Microwave Processor (Milestone, Sorisole, Italy) or the Bond-Max autostainer (Leica Biosystems, Newcastle, UK). The immunohistochemistry markers used were commercially available and were validated in our previous studies (Table 2).

For the antigen-retrieval using the Microwave Processor, the sections were subjected to steaming at 120°C for 5 min in an antigen retrieval solution (10-mM citrate buffer, pH 6.0) (Dako, Glostrup, Denmark). To avoid non-specific targeting, they were treated with serum-free protein blocking solution (Dako, Oslo, Norway) before incubation with the marker of interest. The dilution and incubation conditions were described in Table 2. After incubation, the slides were rinsed twice in saline buffer solution before secondary antibody incubation at room temperature for an hour. For antibody detection, 3, 30-diaminobenzidine (DAB) based detection system (LSAB2; Dako, Norway) was used according to manufacturer's specifications. The stained sections were subsequently counterstained with haematoxylin.

For markers that were stained using the Bond-Max autostainer, the automated immunohistochemical staining process was carried out according to the manufacturer's protocol. Briefly, the sections were subjected to heat-induced antigen retrieval with epitope retrieval ER1 solution (Leica Biosystems) at 100°C for 20 min. Subsequently, they were incubated with the primary antibody for 15 min and were washed subsequently with Bond washing buffer (Leica Biosystems). After washing, the slides were incubated with the secondary antibody (Bond Polymer Refine kit, Leica Biosystems) for 8 min at room temperature. Finally, chromogenic detection of the antibody was achieved by incubation with DAB for 10 min.

### Analysis of immunohistochemistry

**Figure 3:**
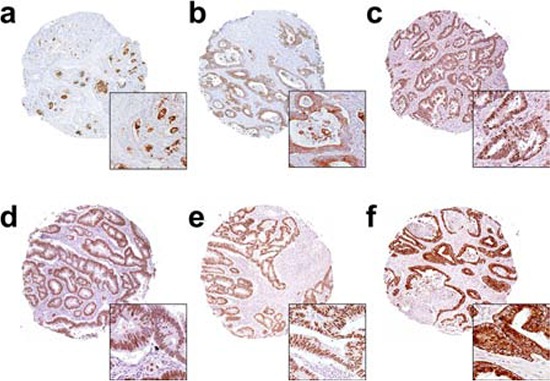
Representative immunohistochemistry images for the markers a. CD133, b. SOX2, c. OCT4, d. P27, e. P53 and f. COX-2

Staining results were assessed independently by CWO and subsequently reviewed by an experienced colorectal pathologist (MST). The scorings were done blinded to the knowledge of patient outcomes and other clinical features. For the markers that expressed immunoreactivity in the nucleus (CD3, CD8, FOXP3, Ki67, p27 and p53), a pathological score was given semi-quantitatively as 0, +1, +2 and +3, corresponding to the percentage of positively stained nucleic (Figure 3). For the cytoplasmic and membrane expressing markers (AXL, CD133, CD44, CK7, CK20, COX2, LGR5, NHE-1, NrCAM, OCT4, SOX2 and STMN1), a four-level semi-quantitative classification corresponding to the staining intensity of the cytoplasmic and membrane immunoreactivity was used (Figure 3).

### Statistical analysis

Only cases with complete information on the immunohistochemical markers were considered for analysis. Relationships between the occurrence frequencies of variables were evaluated by Fisher's exact test. To determine the prognostic value of each variable, univariate Cox proportional hazards regression was carried out. Subsequently, a multivariate Cox model was used to test for independent prognostic value. The data were summarized with hazard ratios (HR) with their 95% confidence intervals (CI). Survival curves were plotted using GraphPad Prism Version 5. The NMF package [30] for the R statistical software [31] was used for the cluster analysis for the immunohistochemical scores. The NMF method allowed identification of clusters in an unsupervised manner based on the Euclidean distance and average linkage. All other statistical analyses were performed using the SPSS package (version 20.0 for Mac, SPSS, USA) with significance set at the 5% level.

## SUPPLEMENTARY FIGURES AND TABLES


