# Supplementary material for: The prognostic value of the stem-like group in colorectal cancer using a panel of immunohistochemistry markers
Source: Oncotarget. 2015 Mar 26;6(14):12763–73. doi: 10.18632/oncotarget.3497 (PMC4494972; doi:10.18632/oncotarget.3497)
Supplement: Supplementary file 1 [file oncotarget-06-12763-s001.pdf]

SUPPLEMENTARY FIGURES AND TABLES

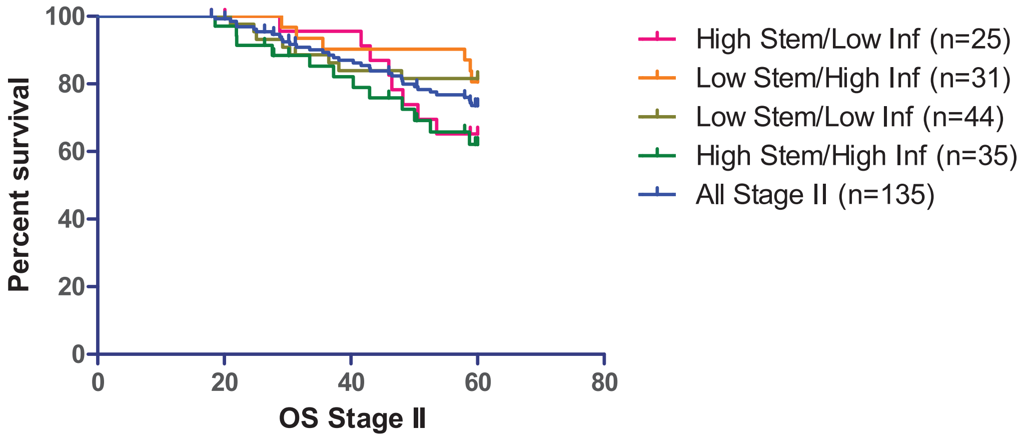

Supplementary Figure S1: Summary of survival differences for different molecular subtypes (MST) in stage 2 CRC cases.

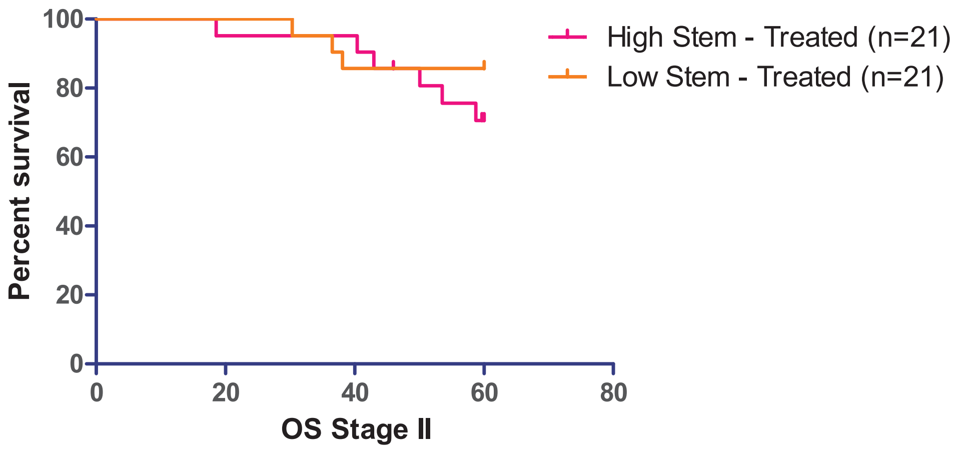

Supplementary Figure S2: Adjuvant therapy did not confer any survival benefits to stage 2 high-stem likes CRC cases.

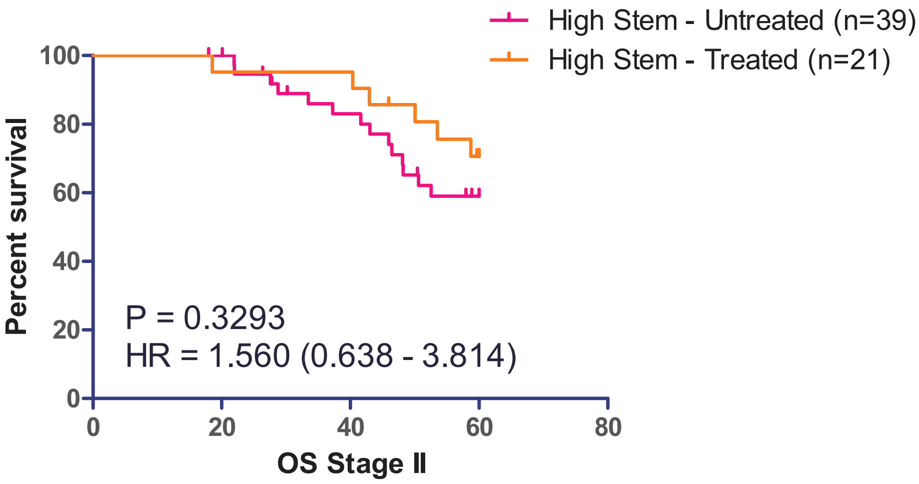

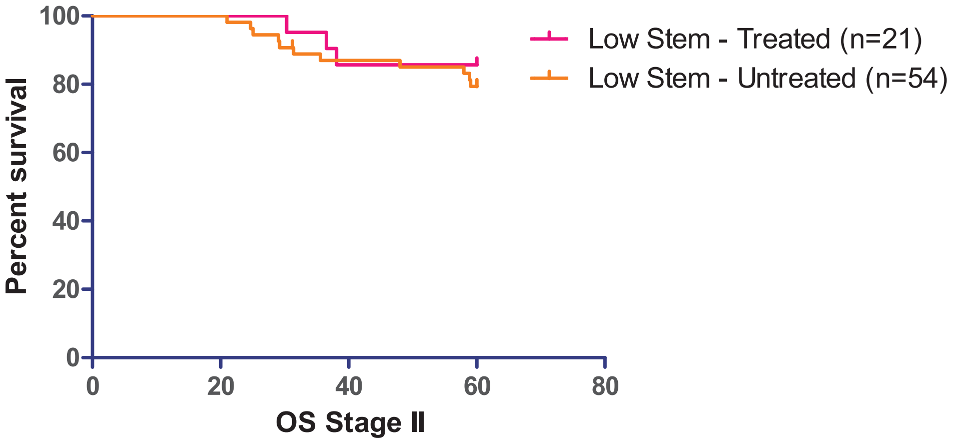

Supplementary Figure S3: High stem-like cases (top figure) showing a trend for treatment benefit in stage 2 CRC cases in contrast to low stem-like cases (bottom figure).

Supplementary Table S1. Summary of differences for survival duration (months) between high stem-like subgroup and low stem-like subgroup across different stages of CRC

Staging	High stem-like group(5 year overall survival)	Low stem-like group(5 year overall survival)	P for log-rank differences
I	80.34	89.80	*NS*
II	61.35	78.49	0.0288
III	42.23	46.85	*NS*
IV	1.94	4.53	*NS*

NS denotes non-significant *p*-values of greater than 0.05.

Supplementary Table S2. Summary of proportions of different MST subgroups of patients within each disease stage

Staging	MST1 (r) High stem-like/ Low inflammatory	MST2 (b) High stem-like/ High inflammatory	MST3 (y) Low stem-like/ High inflammatory	MST4 (g) Low stem-like/ Low inflammatory
I(n = 100)	31	15	13	41
II(n = 162)	30	42	39	51
III(n = 91)	27	33	13	18
IV(n = 140)	15	52	40	33
